# Effects of Different Delignification and Drying Methods on Fiber Properties of Moso Bamboo

**DOI:** 10.3390/polym14245464

**Published:** 2022-12-13

**Authors:** Yifeng Bai, Wenqing Wang, Yongyue Zhang, Xiangwei Wang, Xinzhou Wang, Jiangtao Shi

**Affiliations:** 1 The 38th Institute of China Electronic Science and Technology Group Co., Hefei 230031, China; 2 Department of Wood Science and Engineering, College of Materials Science and Engineering, Nanjing Forestry University, Nanjing 210037, China

**Keywords:** moso bamboo, delignification, drying treatment, performance characterization

## Abstract

Bamboo has become an important kind of fibrous raw material in the world due to its fast-growing property and abundance of natural fiber. During the purification and utilization of bamboo fiber, the removal of lignin is vital and it is affected by the chemical treatment system and drying method. In this paper, the effects of three different delignification chemical systems and three drying methods (air drying, drying and freeze drying) on the physical and chemical properties of bamboo fiber were comparatively studied. The results prove that all three delignification techniques can effectively remove lignin from wood, and by utilizing peroxyformic acid and alkaline sodium sulfite, hemicellulose can be removed to a certain extent. With the selective removal of amorphous hemicellulose and lignin and the hydrolysis of cellulose molecular chains in amorphous regions, all three treatments contributed to an increase in the relative crystallinity of cellulose (ranging from 55% to 60%). Moreover, it was found that the drying methods exerted a certain influence on the mechanical properties of fiber. For instance, drying or air drying would improve the tensile strength of fiber significantly, approximately 2–3.5 times that of original bamboo fiber, and the tensile strength of the drying group reached 850–890 MPa. In addition, the alkaline sodium sulfite treatment had little effect on the thermal stability of bamboo fiber, resulting in high thermal stability of the prepared samples, and the residual mass reached 25–37%. On the contrary, the acetic acid/hydrogen peroxide method exerted great influence on the thermal stability of bamboo fiber, giving rise to a relatively poor thermal stability of prepared fibers, and the residual mass was only about 15%. Among the three drying methods, samples under air drying treatment had the highest residual mass, while those under freeze drying had the lowest. To summarize, the alkaline sodium sulfite method is more suitable for preparing bamboo fiber with higher tensile strength and thermal stability.

## 1. Introduction

Given the numerous environmental concerns, the depletion of fossil fuels and climate change, there has been a raised interest in replacing synthetic fibers in polymer composites with natural plant fibers such as jute, coir, flax, hemp and bamboo [1,2]. These natural plant fibers are widely utilized due to their low density, good thermal insulation and mechanical properties, low price, durability, sustainability and biodegradability [3]. Wood and bamboo, being rich in cellulose, have attracted broad attention from researchers in this field. Bamboo is a kind of fast-growing, sustainable and renewable plant with rich resources. It is a woody fiber material made up of three major chemical compositions, i.e., cellulose, hemicellulose and lignin, which function as the skeleton, matrix and shell, respectively. Being well known for its fast growth rate and naturally high content of solid cellulose crude fiber, bamboo has been proposed as an alternative source of natural fiber.

By different processing methods, bamboo fiber can be processed into raw materials for different industrial applications. For a long time, it has been widely used for construction purposes as well as for pulp and paper in industry [4,5,6]. In particular, for its applications in papermaking, textiles and other fields, chemical methods are usually required to remove lignin in bamboo. Therefore, the removal of lignin is considered to be crucial in the preparation and utilization of bamboo fiber. Commonly used delignification methods include the sodium chlorite method, the alkaline sodium sulfite method and the performic acid method [7,8,9]. Additionally, the drying process will have an impact on the morphology and mechanical strength of fibers. For instance, freeze drying can better preserve the morphology of submicron fibers and reduce fiber shrinkage. However, when treated with air drying, the separated large fibers will produce capillary tension, causing the nanofibers that make up the fibers to collapse together. Thus, the increased capillary force will be combined with hydrogen bonding force, contributing to the collapse of cellulose nanofibrils and the self-densification of the corresponding large fibers, and thereby forming a more stable structure [10].

Although much research has been conducted on the functional transformation for delignified materials of wood and bamboo, there are limitations with respect to the research on changes in properties of the materials themselves after delignification. Different delignification methods will have different influences on the morphology and properties of bamboo fiber. By adopting three different delignification processes and three drying methods (i.e., air drying, drying and freeze drying), we comparatively studied the physical and chemical properties of bamboo fiber under different treatment methods. This paper provides effective data for the processing and utilization of bamboo resources in the future, which is of great significance to improve the utilization rate of bamboo.

## 2. Materials and Methods

### 2.1. Raw Material

The natural bamboo (3–5 years of age) was collected from Nanjing Forestry University, Nanjing, Jiangsu Province. Hydrogen peroxide, concentrated sulfuric acid, sodium hydroxide and anhydrous sodium sulfite were purchased from Nanjing Chemical Reagent Co., Ltd. (Nanjing, Jiangsu, China). Formic acid and acetic acid were purchased from Sinopharm Chemical Reagent Co., Ltd. (Nanjing, Jiangsu, China). Deionized water (DI) was prepared in the laboratory.

### 2.2. Delignification Treatment

The original bamboo was cut into slices of different lengths (excluding joints). The green parts on the surface were scraped off and cooked in boiling water for 1 h to remove water-soluble organic substances and trapped air. Subsequently, three different delignification methods were adopted. Below, we introduce their corresponding processes. The first method was the peroxyformic acid method. The bamboo chip was delignified at 50 °C for 12 h with the prepared peroxyformic acid solution (the molar ratio of 30% hydrogen peroxide and formic acid was 1:1, and 1 wt % sulfuric acid was added as catalyst); then, 0.5 wt % sodium hydroxide solution was used for neutralization for 5 to 10 min, and finally the chip was washed in deionized water several times to remove chemicals. The second method was the hydrogen peroxide/acetic acid method. This was a process to impregnate the bamboo chip into a mixed solution of hydrogen peroxide and acetic acid with a molar ratio of 1:1, treat it in a 100 °C water bath for 6 to 8 h and wash it with deionized water multiple times to remove chemicals. The last method was the alkaline sodium sulfite method. The treated bamboo chip was impregnated in a mixed solution of 4 mol/L NaOH and 2.5 mol/L Na_2_SO_3_; we conducted lignin removal at 80 °C for 6 to 8 h, then bleached with 30% H_2_O_2_ and washed with deionized water several times to remove chemicals.

### 2.3. Drying Treatment

The samples derived from the above three delignification processes were treated with three different drying methods, namely air drying, drying and freeze drying. In air drying, the treated bamboo was first placed at room temperature for 24 to 48 h, and the bamboo fiber was then obtained by manual stripping. In the drying process, the bamboo was placed in an oven at 60 °C for 6 to 8 h. In freeze drying, the treated bamboo was frozen in a −15 °C refrigerator for 24 h until it was completely frozen, and afterwards, it was put in a freeze drying machine for 24 to 48 h. After performic acid treatment, the samples obtained by the three drying processes were numbered A1, A2 and A3, respectively. Similarly, those treated with acetic acid/H_2_O_2_ and the three drying processes were B1, B2 and B3, and those treated with alkaline sodium sulfite and the three different drying processes were C1, C2 and C3.

### 2.4. Characterization

The morphology of the samples was measured by a field emission scanning electron microscope (TM-1000), and bamboo fibers were prepared into fiber bundles with a length of approximately 2 mm and treated with metal spraying.

Approximately 1 mg bamboo fiber powders from 4 samples (drying natural bamboo and bamboos treated with three different delignification methods) were analyzed using a Fourier Transform Infrared (FTIR) spectrometer (VERTEX 80 V). The spectrometer was adjusted to mid-infrared transmission mode (KBr), and FTIR images were obtained in the mid-IR light region range from 4000 to 500 cm^−1^. The Ultima IV X-ray diffractometer was utilized to study the cellulose crystallinity of natural bamboo (CK) and nine groups of fiber samples. The settings were as follows. X-ray tube: copper target, tube voltage: 35 kV, tube current: 25 mA, X-ray diffraction patterns: 2 *θ*/*θ* scan, scanning range: 2*θ* = 5~45°, sampling interval: 0.05°. We calculated the value of relative crystallinity for cellulose by Turley’s empirical method. The formula was as follows: C_r_I = (*I_002_* − *I_am_*)/*I_002_* × 100%, where C_r_I is the relative crystallinity of cellulose, *I_002_* is the maximum intensity of (002) lattice diffraction angle—i.e., the diffraction intensity of the crystalline region—and *I_am_* is the scattering intensity of 2*θ* = 18° amorphous background diffraction.

The processed samples were separated into bamboo fibers with a length of 50 mm and a diameter of 0.25–0.35 mm. We used a universal tensile machine (SHIMADZU Instruments, Kyoto, Japan) at a loading force 5 KN at a speed of 5 mm/min to test the tensile strength of CK and the nine groups of fiber samples. The thermal degradation characteristics were determined by a thermal gravimetric analyzer (Netzsch TG209 F3 Nevio, Selb, Germany). The settings were as follows. Experimental temperature: 30 °C to 600 °C, linear heating rate: 10 °C/min^−1^, nitrogen flow rate: 10 mL/min, weight of each samples: approx. 5 mg.

## 3. Results and Discussion

### 3.1. Microstructure

**Figure 1:**
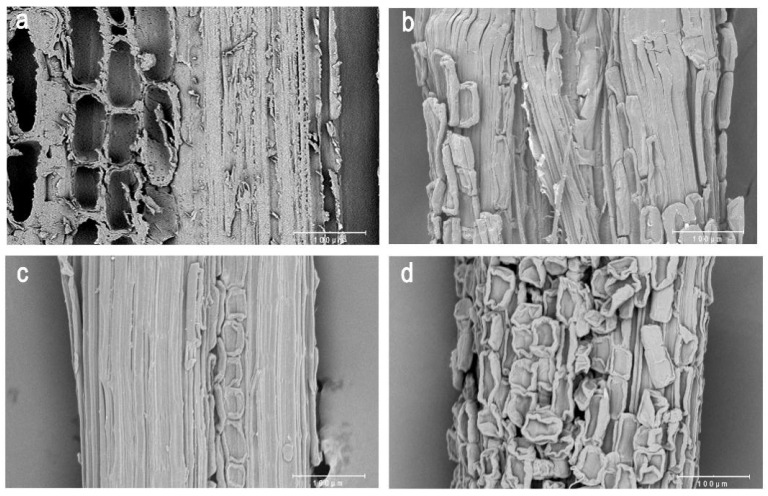
SEM images of natural bamboo (**a**) and bamboo fibers with three different delignification methods (**b**–**d**). (**b**) Peroxyformic acid method; (**c**) acetic acid/dioxygen water method; (**d**) alkaline sodium sulfite method. Bar = 100 µm.

The microstructure changes in bamboo during chemical delignification were studied by scanning electron microscopy (SEM) (Figure 1). In natural bamboo, fibers with thick wall characteristics are closely combined with hollow thin-walled cells. However, after delignification in different ways, the parenchyma cells shrunk and detached to different degrees. Bamboo treated with peroxyformic acid (Figure 1b) retained a certain amount of parenchyma cells and attached to the surface of fiber bundles. Most parenchyma cells of bamboo treated by acetic acid/dioxygen water (Figure 1c) had fallen off. In contrast, most parenchyma cells of bamboo treated with the alkaline sodium sulfite method (Figure 1d) remained on the surface of fiber bundles, which may have been caused by incomplete lignin removal by this method. In a word, after delignification treatment, the fiber bundles of the three kinds of bamboo retained their natural structure, without obvious damage.

### 3.2. Chemical Functional Groups

**Figure 2:**
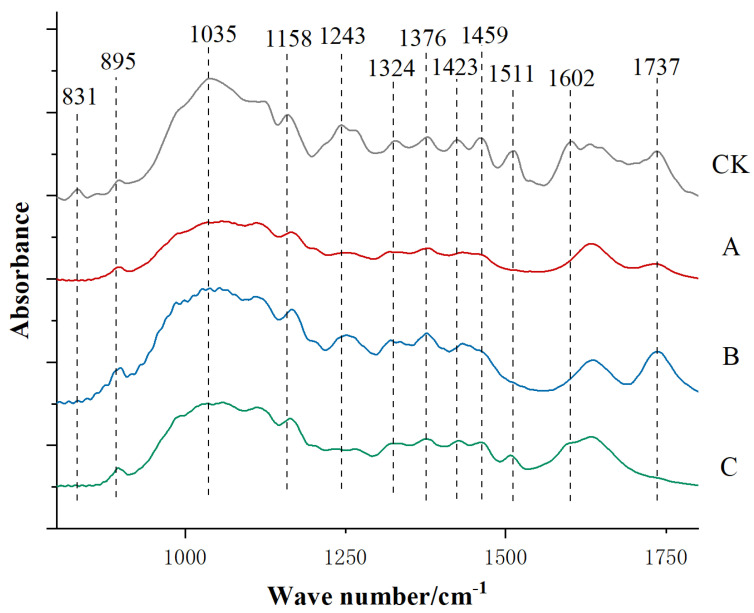
FTIR diagram for bamboo fiber of natural bamboo (CK) and delignified bamboos (A, B, C).

In order to further study the chemical composition changes in bamboo fibers after three different delignification processes, the FTIR results for bamboo fibers of natural bamboo and bamboos treated with three different delignification methods were compared and analyzed. The area which ranged from 500 cm^−1^ to 1800 cm^−1^ comprised 12 absorption vibrational bands (Figure 2, Table 1). The vibrational bands at 1602 cm^−1^, 1511 cm^−1^ and 1423 cm^−1^ were assigned to the vibration of the C=C unsaturated bonds of the aromatic skeleton in lignin. The one at 1459 cm^−1^ was attributed to the asymmetric bending of lignin CH^3^. The absorption vibrational band at 1243 cm^−1^ arose from the clove ring and C–O stretching in lignin and xylan. After three different delignification treatments, the absorption vibrational band intensity at 1602 cm^−1^, 1511 cm^−1^, 1459 cm^−1^, 1423 cm^−1^ and 1243 cm^−1^ underwent varying degrees of weakening or even disappearance, which demonstrated that lignin decomposition had occurred during the delignification processes, and alkaline sodium sulfite treatment (Line C on Figure 2) was found to be relatively weak in delignification. In addition, the vibrational band at 831 cm^−1^, which was assigned to C–H vibrations in Guaiac-based derivatives, disappeared completely after delignification treatment.

Apart from the removal of lignin, the vibrational band intensity of cellulose at 1376 cm^−1^, 1324 cm^−1^, 1158 cm^−1^ and 895 cm^−1^ went through a small change after delignification treatment. Comparing the hemicellulose vibrational band intensity at 1737 cm^−1^ and 1035 cm^−1^, it could be found that the delignification treatments of peroxyformic acid (A) and alkaline sodium sulfite (C) had a certain degree of influence on hemicellulose, which might lead to hemicellulose degradation [14], whereas acetic acid/hydrogen peroxide (B) had less of an effect on hemicellulose [15].

### 3.3. Crystalline Structure

**Figure 3:**
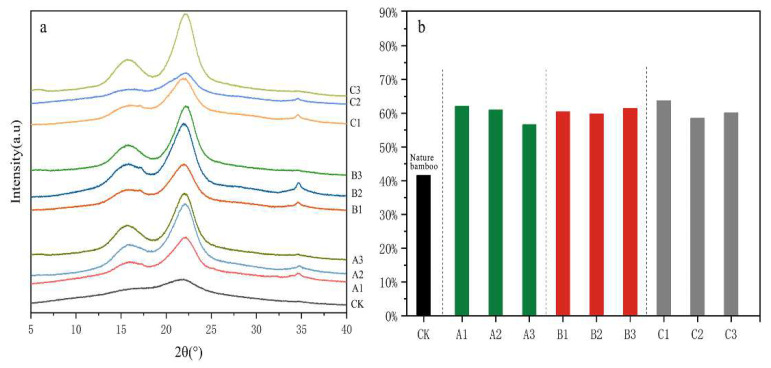
(**a**) X-ray diffraction (XRD) patterns of natural bamboo fibers (CK) and bamboo fibers treated with different delignificati on processes. (**b**) The crystallinity values of natural bamboo fibers (CK) and bamboo fibers treated with different delignification processes.

Cellulose is one of the main components of bamboo and makes up 40% to 60% of the chemical composition of bamboo. Using an X-ray diffractometer, the molecular chains of cellulose with high crystallinity can be detected, contributing insight into the effects of different treatments on crystalline and amorphous regions of cellulose. In this study, an X-ray diffractometer was used for the analysis of the original bamboo fibers and those treated with different chemical processes, and the results are shown in Figure 3.

The three typical peaks corresponding to the (040), (002) and (101) lattice planes of cellulose were 34.58°, 22.12° and 15.72°, respectively, among which the peak of the (002) plane reflected the width of the crystalline region, while that of the (040) plane represented the length [16]. The intensity of the (040) peak is low, and the characteristic peaks at (101) and (002) are used to reflect the crystal structure [17]. The positions of characteristic peaks for the original bamboo fibers and the delignified ones were basically the same, suggesting that different delignification treatments had little effect on the crystalline region of cellulose. However, the (101) and (002) peak intensities of three delignified bamboos are higher than those of the original bamboo. The crystallinity of the original bamboo was about 40%, while after three different delignification treatments, the crystallinity values increased significantly (ranging from 55% to 60%). The reason for this phenomenon was mainly as follows: during the delignification process, the amorphous hemicellulose and lignin were selectively removed. Meanwhile, the cellulose molecular chains in the amorphous region were hydrolyzed, which resulted in the exposure of hydroxyl groups on the surface. Coupling with microfibers on the surface of the crystalline region, the process facilitated the formation of hydrogen bonds, thereby improving crystallinity. Nevertheless, different drying methods had inconspicuous effects on the crystallinity of cellulose.

### 3.4. Mechanical Properties

**Figure 4:**
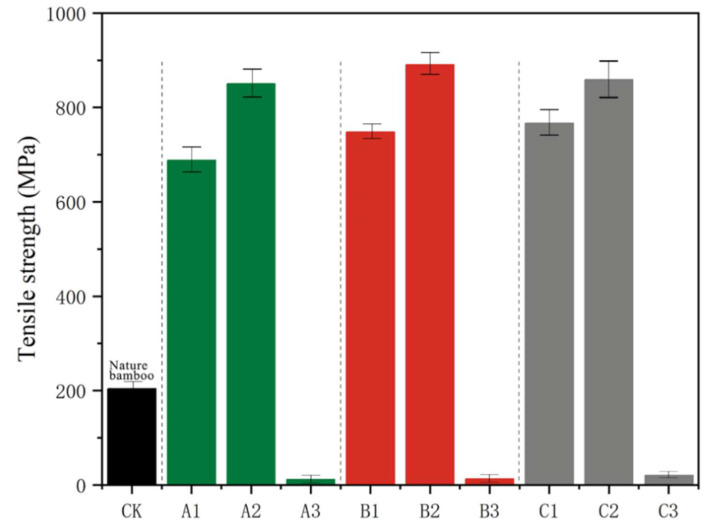
Tensile strength of natural bamboo fibers (CK) and bamboo fibers treated with different delignification processes.

Figure 4 shows the tensile strength of the original bamboo without delignification (about 200 MPa), and that for the nine groups of samples treated with three delignification processes and three drying methods. Each group of samples was tested for tensile strength five times. Compared with samples treated with the other two drying methods, the tensile strength of the samples processed by freeze drying was much lower (at 11–19 MPa), which was consistent with the characteristics observed in the macroscopic state. This might be attributed to the fact that the removal of the cell wall matrix in the freeze drying state destroyed the integrity of the original cell wall, resulting in a large number of interstitial structures on the fiber surface, thereby significantly reducing the tensile strength of the fiber [18]. Compared with the samples of original bamboo, the tensile strength of the ones treated with drying and air drying increased by 2 to 3.5 times. In addition, the ones treated with drying, compared with the ones treated with air drying, increased by an average of 21% in their tensile strength values (at 850–890 MPa), which was consistent with the results derived from previous research [19]. The underlying reason for this phenomenon is that compared with samples treated with drying, the ones treated with air drying had higher moisture contents, and with the increase in moisture content, the stiffness of hemicellulose and residual lignin was significantly reduced. As a result, the connection between the microfibrils and the matrix was no longer as tight as before, and when external forces were exerted to the cell wall, slippage occurred between microfibrils, which resulted in an increase in the tensile strain of fibers, thereby reducing the strength of the fibrous cell wall (which was manifested as a decrease in tensile strength). Under the same drying condition, different delignification methods had less of an effect on tensile strength, and the samples delignified with peroxyformic acid (A) exhibited lower tensile strength than the samples treated with the other two methods, the reason for which might be that hemicellulose, which plays an interface role in connecting cellulose and lignin in the cell wall, was partially degraded, and such removal of hemicellulose directly contributed to the destruction of the integrity of the cell wall, and hence, the ability to resist external damage was reduced [20]. This inference corresponds with the results of FTIR in the preceding part. Sergejs et al. showed that the tensile strength of fiber bundles can be significantly improved by adding an appropriate amount of xylan (the main component of hemicellulose) to cellulose nanopapers, which is similar to the results in this paper [21].

### 3.5. Thermal Properties

**Figure 5:**
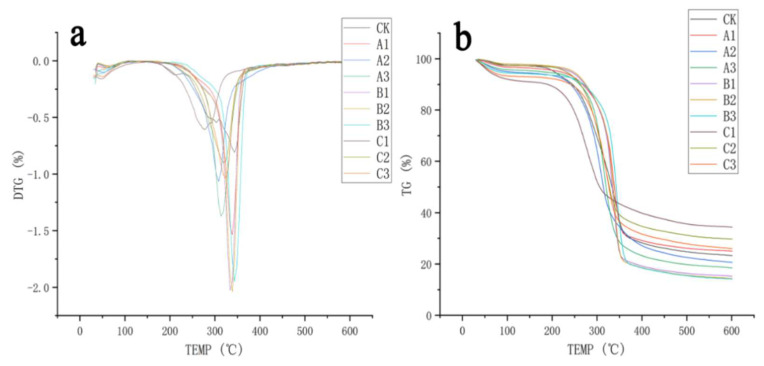
(**a**) Derivative thermogravimetry (DTG) and (**b**) thermogravimetric analysis (TGA) curves of different bamboo fiber samples.

In order to study the influence of different delignification and drying methods on the thermostability of bamboo, TGA and DTG curves of samples were analyzed and are shown in Figure 5. It can be learned from the DTG curves of the CK group that the thermal degradation of bamboo fiber can be divided into three stages [22]. In the first stage (30–150 °C), when the mass decrease was mainly caused by the evaporation of water, only the C2 group had a mass decrease of about 9%, which probably resulted from the high initial moisture content, and the mass loss rates of the remaining nine groups of samples presented little difference. In the second stage (250–300 °C), when hemicellulose began thermal degradation, the CK groups exhibited obvious mass loss, and among the nine groups of delignified samples, only the C2 group had a large amount of mass loss. It may be that there are many parenchyma cells attached to the fiber bundle—parenchyma cells contain more hemicellulose than fiber cells, so the maximum thermal degradation temperature (T_max_) appears at this stage [23]. The third stage (300–350 °C) was the thermal degradation of cellulose. Since the CK group had the highest cellulose content in bamboo fiber, the highest mass loss rate occurred at this stage. After the removal of lignin, the eight groups of delignified samples (except group C2) had larger proportions of cellulose, thus presenting higher mass loss rates, and T_max_ values were all within this range. Moreover, among the delignified samples, the samples treated with alkaline sodium sulfite had the highest residual mass, approximately 25–37%, which might result from the weak lignin removal effect of the alkaline sodium sulfite method and the high proportion of lignin with a wide temperature range of thermal degradation (160–900 °C). This assumption was confirmed by the FTIR results described earlier in this paper. Compared with the peroxyformic acid method, acetic acid/hydrogen peroxide had a higher content of hemicellulose, making it easy to facilitate thermal degradation and a higher removal degree of lignin, which has superior thermal stability. Therefore, the residual mass after thermal degradation was lower, only about 15% [24]. Among the three drying groups, the air drying group had the highest residual mass, and the freeze drying group had the lowest.

## 4. Conclusions

We comparatively studied the effects of three delignification processes and three drying methods on the properties of bamboo fiber. On one hand, different delignification processes, namely peroxyformic acid, acetic acid/hydrogen peroxide and alkaline sodium sulfite, exerted obvious effects on the thermal stability of bamboo fiber, but there was no significant difference in the mechanical properties between the samples of the three delignification methods. To be specific, in FTIR spectra, the characteristic peaks of lignin at 1511, 1423 and 1243 cm^−1^ underwent various degrees of weakening or even disappearance, suggesting differences in lignin removal, and the alkaline sodium sulfite method was less effective in removing lignin. Similarly, different treatments also contributed to different degradation rates of hemicellulose: peroxyformic acid method and alkaline sodium sulfite methods caused the partial removal of hemicellulose. XRD analysis proved that the three delignification methods had little influence on the crystallinity of cellulose, and after delignification, the relative crystallinity of all bamboo fibers had an obvious increase of about 20%. In addition, the tensile strength of the freeze drying samples was only 5.5–9.5% of that of the natural bamboo fibers, but the tensile strength of the samples treated with drying and air drying increased by 2 to 3.5 times. The three delignification methods also had an effect on the thermal stability of bamboo fiber, among which samples treated with alkaline sodium sulfite had the best thermal stability and the highest residual mass (25–37%). On the other hand, the drying methods produced inconspicuous effects on crystallinity, but had a great influence on the mechanical strength of bamboo fiber. Freeze drying could reduce the tensile strength of the fiber, whereas air drying and drying improved it. Samples treated with drying had the highest tensile strength (850–890 MPa), while the tensile strength of the samples processed by freeze drying was much lower (11–19 MPa). For thermal stability, the air drying group had the highest residual mass, while the freeze drying group had the lowest.

## Figures and Tables

**Table 1:** Assignment of FTIR spectra absorption peaks of natural bamboo [11,12,13].

Wave Number (cm^−1^)	Functional Group	Assignment
1737	C=O	non-conjugated C=O (xylan) in hemicellulose
1602	C=C	C=C unsaturated bonds, aromatic skeleton vibration in lignin
1511	C=C	aromatic skeleton vibration in lignin
1459	C–O, C–H	asymmetric bending in CH3 (lignin)
1423	CH_2_	aromatic skeleton vibration (lignin) and plane C–H deformation (cellulose)
1376	C–H	C–H deformation in cellulose and hemicellulose
1324	O–H	phenol group (cellulose)
1243	C–O	clove ring and C–O stretching in lignin and xylan
1158	C–O–C	C–O–C vibrations in cellulose and hemicellulose
1035	C–O, C–H	C–O stretching in cellulose and C–H stretching of hemicellulose in lignin
895	C–H	C–H deformation in cellulose
831	C–H	C–H vibrations in Guaiac-based derivatives

## Data Availability

The data presented in this study are available on request from the corresponding author.
